# Unilateral lateral mass fixation of cervical spinal low-grade chondrosarcoma with intralesional resection: A case report

**DOI:** 10.3892/ol.2014.1956

**Published:** 2014-03-07

**Authors:** BO CHEN, YAN YANG, LIANG CHEN, FENG ZHOU, HUILIN YANG

**Affiliations:** Department of Orthopedic Surgery, First Affiliated Hospital and Orthopedic Institute, Soochow University, Suzhou, Jiangsu 215007, P.R. China

**Keywords:** lateral mass fixation, chondrosarcoma, intralesional resection, cervical spine

## Abstract

In total, ~10% of chondrosarcomas arise from the mobile spine, and these are prone to local recurrence despite being low-grade malignant tumors. Almost all patients will present with pain and a palpable mass in the area of the lesion. For adequate management of the disease, an early diagnosis and careful surgical staging are important. The present study reports a case of cervical spinal low-grade chondrosarcoma in a young female presenting with a slow-growing mass that had not metastasized during a 3-year period. A unilateral lateral mass fixation system of screws and rods was installed following an intralesional resection of the tumor. At present, two years following the surgery, the patient exhibits no neurological deficiency symptoms. Therefore, unilateral fixation presents an effective alternative technique for the treatment of patients with a lesion on the cervical spine.

## Introduction

Chondrosarcoma is a rare disease, with an estimated incidence of 1 in 200,000 per year (1), and occurring predominantly in the extremities. Approximately one-third of chondrosarcomas occur in the spine (2). Conventional radiation therapy and chemotherapy have not been proven to be effective in chondrosarcoma treatment (3,4), and surgical resection remains the standard method (5–7). Chondrosarcomas grow slowly and rarely metastasize, and they have an excellent prognosis following en bloc resection (8). For the present patient, performing en bloc resection was impossible, as the tumor infringed on important vascular and nervous tissue. The current study presents a case of cervical spinal low-grade chondrosarcoma in which the installation of a unilateral fixation system provided cervical spinal stability following an intralesional resection of the low-grade chondrosarcoma. Written informed consent was obtained from the patient for participation in this study.

## Case report

**Figure 1:**
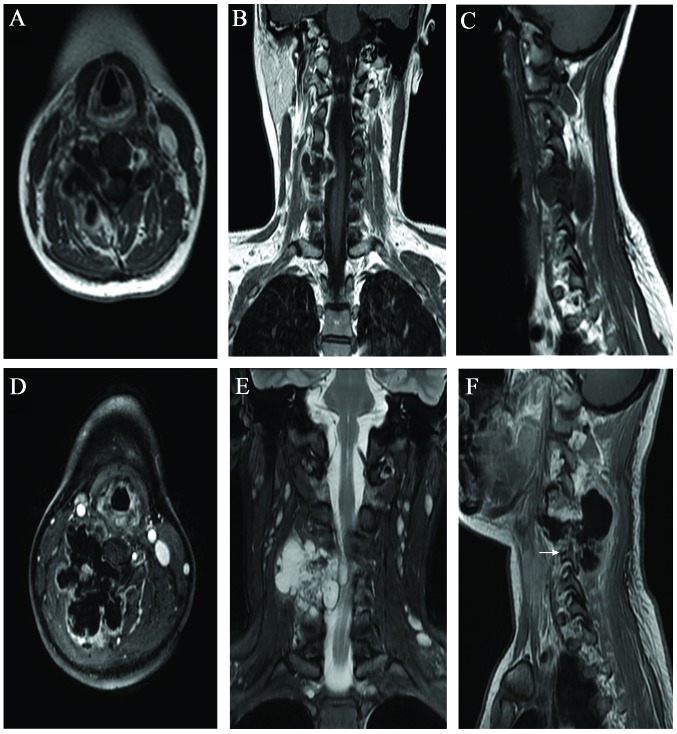
(A) Axial, (B) coronal and (C) sagittal T1-weighted images captured 3 years prior to the palpable mass of the cervical spine. (D) The coronal view, Gd-DTPA-enhanced image, showing soft-tissue expansion and the destruction of the adjacent normal tissues, including the right side of the processus transversus, lamina and spinous process. (E) The fat-suppressed image discloses a tumor lesion, and compression of the right side of the spinal cord. (F) The sagittal T1-weighted image demonstrates that the nerve root was being squeezed (white arrow). Gd-DTPA, gadopentetic acid with diethylenetriaminepentacetate.

A 29-year-old female was admitted to the Department of Orthopedic Surgery (First Affiliated Hospital and Orthopedic Institute, Soochow University, Suzhou, Jiangsu, China) presenting with complaints of increasing pain at the back of the neck, with a mass on the cervical spine and right upper extremity weakness for the previous 2 months. The patient initially presented to the hospital 3 years prior to this with soreness in the right side of the neck lasting for 12 months. The diagnosis from an incisional biopsy was of a low-grade chondrosarcoma of the cervical spine (Fig. 1A–C).

**Figure 2:**
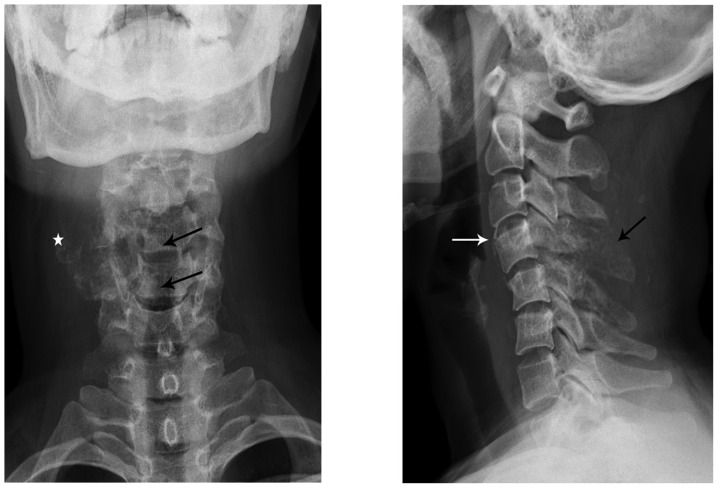
Antero-posterior and lateral view of the radiograph revealing the destruction of the spinous process of C4 and C5 (black arrow), and a soft tissue mass with calcification (pentagram). The front of the vertebral body had been infiltrated by the tumor (white arrow).

**Figure 3:**
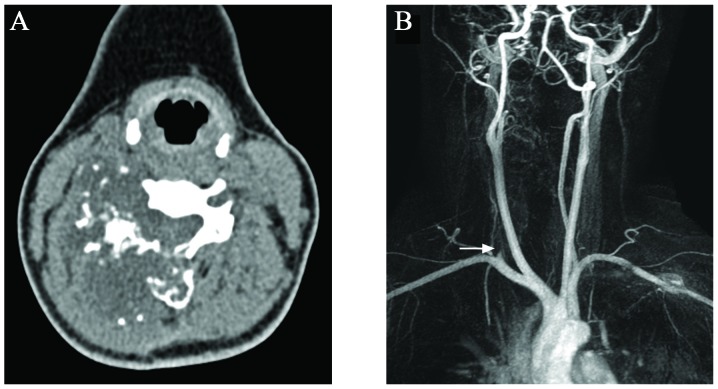
(A) Axial CT scan of the C5 vertebra. There is a defined lytic lesion and a large soft-tissue mass within the mottled calcification, and the tumors that were involved in the processus transversus, lamina and spinous process are on the right. (B) Angiography showing a lack of blood flow in the right vertebral artery (white arrow) and compression of the common carotid artery. CT, computed tomography.

A physical examination showed that there was weakness in the upper right extremity and hypermyotonia in the lower extremities. Upon admission, plain radiography, computed tomography (Figs. 2 and 3) and magnetic resonance imaging (Fig. 1D–F) demonstrated an expansive mass lesion. Computed tomography scans of the chest, abdomen and pelvis identified no other lesions. Angiography (Fig. 3B) was performed for the lesions that were close to the vertebral artery to evaluate the displacement and involvement of the vessels.

**Figure 4:**
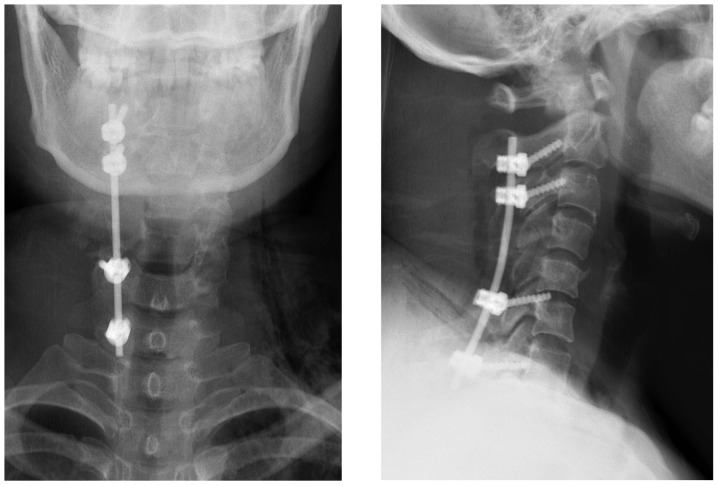
Post-operative anteroposterior and lateral radiographs.

**Figure 5:**
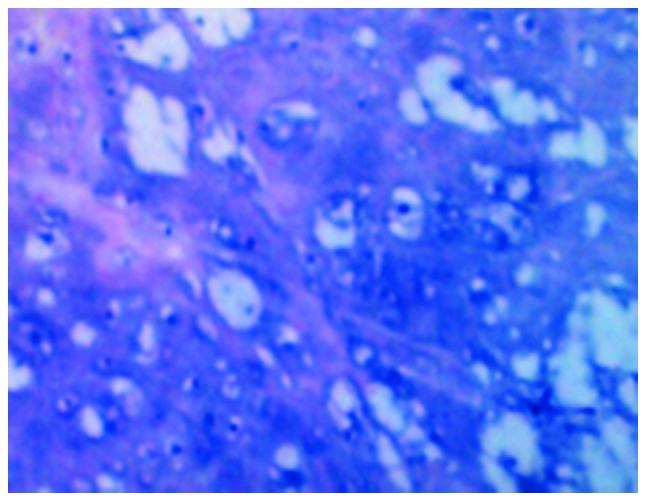
Well-differentiated conventional chondrosarcoma composed of atypical chondrocytes.

**Figure 6:**
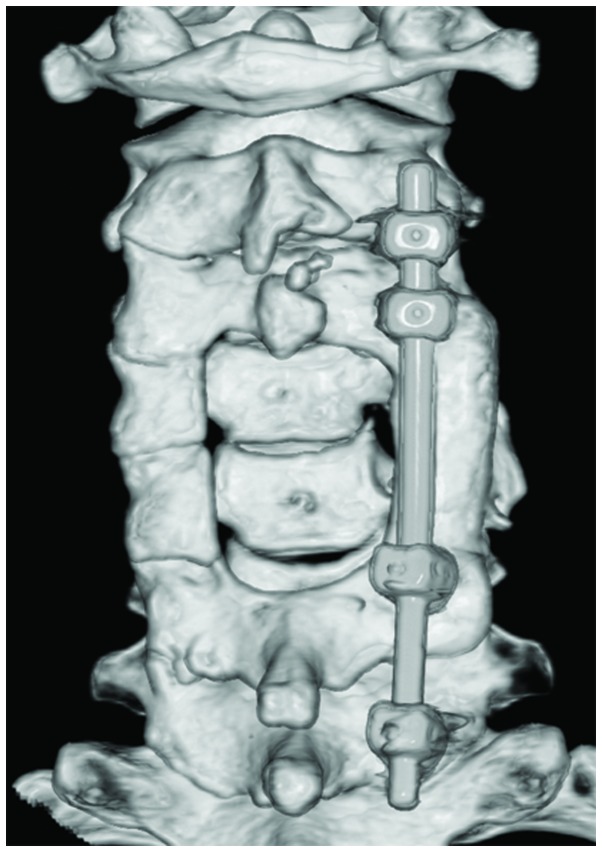
A 3-dimensional reconstruction of the CT scan showing successful fusion at 2 years post-surgery. CT, computed tomography.

Embolizations were performed through digital subtraction arteriography prior to the surgery. In the present case, the mass had become involved with the spinal canal and had wrapped around the vertebral artery and adhered to the root of C4/C5 (Enneking stage IB and Weinstein-Boriani-Biagini stage 8–12/A–D). Therefore, en bloc resection with a tumor-free margin could not be achieved. The patient received posterior surgery only, involving piecemeal removal of the tumors, until tumor-free margins were obtained. The bilateral arcus vertebrae and the right articulationes zygapophysiales of C4/C5 were removed and part of the spinous process of C3 was excised. A fixation system of screws and rods was installed (Fig. 4) and the axis rods were bent to match the cervical curvature. Bone harvested form the posterior superior iliac spine was inserted as a bone graft at the site of the lesion. Post-surgery, the patient underwent radiotherapy (five days a week at a dose of 48 Gy for five weeks), and histopathological examination diagnosed a low-grade chondrosarcoma (Fig. 5). The post-operative neurological symptoms improved and therefore the patient was discharged 10 days after the surgery. In the 2 years following the surgery the patient had no neurological deficiency symptoms and only a mildly uncomfortable left cervical spine (Fig. 6).

## Discussion

Pain is the most common symptom of chondrosarcoma, and another is a palpable mass. Neurological deficits have also been reported in half of all affected patients (9). The pain is often insidious in nature and can be present for weeks to years (10). Low-grade chondrosarcomas grow slowly and rarely metastasize (8,11). In the 3 years previous to the present study, the patient did not receive surgery, chemotherapy or radiotherapy. During the 3 years, the tumor grew by expansion to compress the spinal cord and nerve roots and destroy the adjacent normal tissues, including the right side of the articulationes zygapophysiales, lamina and spinous process, without metastasizing.

In general, with the current treatment strategies, including local adjuvant therapy, the local recurrence rate is low for low-grade chondrosarcoma, and there is a decrease in morbidity, but recurrence can occur 10 years post-surgery (8). Recurrence of chondrosarcoma is usually within 3–5 years post-surgery. If a subtotal excision resection is performed and not en bloc resection, then the recurrence occurs sooner (5,12). Low-grade chondrosarcoma can be safely treated by an extended intralesional excision in the extremity; the long-term clinical results of this method are promising and there is satisfactory local control (5,8,13,14). Riedel *et al* (13) reported that there has been a trend of moving away from the use of wide resection in select low-grade tumors, as a combination of a low metastatic potential and a low local recurrence rate has been noted for the intralesional surgery of low-grade chondrosarcoma. No difference in the overall survival rate between the intralesional curettage group and the wide resection group in patients with conventional low-grade chondrosarcoma of the long bones has been reported (15).

However, patients with lesions in the axial skeleton have the worse prognosis when treated with intralesional resection (5–7,11,16). The results from these studies may be in correlation with the complex spinal anatomy, which leads to difficulty in the thorough excision of tumors and inadvertent intraoperative contamination (5,17). For treatment of spinal chondrosarcoma, surgery is critical; it should aim to preserve and possibly improve the functionality of the spine, and to relieve pain and control local tumor recurrence, which in turn promises a longer survival rate (18).

En bloc resection with wide or close margins remains the best oncological management of the spine (3,5–8,16). A long-term survival rate has only been observed for low-grade spinal chondrosarcoma in patients treated with repeated intralesional excisions of the recurrent disease, combined with radiation therapy (19). Thus, an en bloc resection is the current recommendation for numerous primary tumors of the thoracic, lumbar and sacral spine (17). Owing to its proximity to vital neurovascular structures and combined with the complex spinal anatomy, chondrosarcoma of the spine poses difficulties with regard to the surgical procedures performed (7), and the majority of these lesions cannot be excised in an ideal en bloc manner. If en bloc resection is recommended for a patient, the high rate of surgical morbidity and potential functional impairment must be weighed against intentional tumor transgressions for functional sparing and the consequences of tumor-margin violation (16). Virkus *et al* (20) demonstrated that the potential loss of function must be seriously considered, as it has not been definitively shown that local recurrence has an effect on the overall patient survival rate. In the present patient, performing an en bloc resection was impossible since the tumor was wrapped around the right vertebral artery and was adherent to the nerve root, therefore, an intralesional tumor resection was performed.

The goal of this surgical procedure was adequate neurological recovery. When laminectomy is required for spinal cord compression, the possibility of future spinal instability must be assessed at an early stage (21). It has been reported that lateral mass screw fixation is a safe and effective technique for stabilization (22). Chen *et al* (23) demonstrated that unilateral fixation is good enough to maintain the stability of the device-spine construct through an *in vitro* biomechanical study. The clinical outcome and lumbar fusion rate in the unilateral pedicle screw fixation were almost identical with those in the bilateral method, as reported by Suk *et al* (24). In comparison to the bilateral variable screw placement model, Goel *et al* (25) concluded that the unilateral system was more likely to reduce the stress shielding of the vertebral bodies and was less rigid. In the present patient, the disc was intact without degeneration and the left articulationes zygapophysiales was undamaged. We hypothesized that unilateral lateral mass fixation was sufficient to reconstruct the stability of the spine.

In accordance with the requirements of the patient, the unilateral fixation was performed. Xue *et al* (26) reported that the unilateral pedicle screw instrumented transforaminal lumbar interbody fusion neither decreased the fusion rate nor increased the complication rate. The present patient felt only mild discomfort in the left cervical spine and has so far been disease-free for 2 years post-surgery.

Unilateral fixation can provide spinal stability, although the mechanical characteristics of the local change are unknown, which may cause patients to feel uncomfortable. If unilateral cervical fixation can provide enough stability, it may present as a possible treatment option for unilateral cervical spine injury caused by trauma. A clinical study is required to determine the biomechanical characteristics of unilateral fixation. As the patient follow-up time of this condition is not long enough, the long-term effect remains to be observed.

For curative intentions, doses of >60 Gy are required to achieve local control (8). Harwood *et al* (27) indicated the necessity of delivering a dose of >50 Gy in order to have an impact on chondrosarcoma. Great care must be taken in planning the volume applied to tumors arising in the sacrum, vertebrae or ribs close to the vertebral colon. For tumors that have spread into the spinal canal, a dose of 48 Gy should be chosen.

In conclusion, chondrosarcoma is generally a low-grade, slow-growing tumor, which rarely metastasizes. Low-grade chondrosarcoma can be safely treated with intralesional curettage without increasing the risk for local recurrence or metastatic disease in the extremities. Pre-operative planning for surgical tumor removal and spine stabilization is mandatory. Although unilateral lateral mass fixation can provide spinal stability, determination of the biomechanical characteristics is required in future studies.
